# Effectiveness of targeted financial aid on disability welfare for the ageing population in China: A quasi-experiment study

**DOI:** 10.7189/jogh.14.04222

**Published:** 2024-10-25

**Authors:** Hongchuan Wang, Zhe Chen, Kaibo Xu, Wannian Liang

**Affiliations:** 1 School of Public Policy and Management, Tsinghua University, Beijing, China; 2 Institute for Contemporary China Studies, Tsinghua University, Beijing, China; 3 School of Politics and Public Administration, Soochow University, Suzhou, China; 4 Vanke School of Public Health, Tsinghua University, Beijing, China; 5 Institute for Healthy China, Tsinghua University, Beijing, China

## Abstract

**Background:**

Addressing the problem of disabilities and disability deterioration is a key task for healthy ageing. Financial aid has been an effective measure for vulnerable groups, especially ageing people with disabilities. However, the effects of targeted financial aid on preventing disability deterioration remain unknown. The Chinese government launched a targeted financial aid programme aimed at people with disabilities. In this study, we investigated the causal effects of such targeted financial aid on disability deterioration prevention for elderly people with disabilities in China.

**Methods:**

The data set used in this study included 36 640 elderly individuals with disabilities in China between 2016–19. We constructed a quasi-experiment approach and used a difference-in-differences (DID) method to examine the counterfactual differences between the treatment group in four cities that implemented such targeted financial aid in 2018 and the control group in three cities that did not adopt the policy over the study period. We employed propensity score matching (PSM) jointly with DID to mitigate selective bias. For sensitivity analysis, we conducted supplementary analyses on alternative samples, focusing on each of the treated cities respectively. Besides the main outcome, we also used fixed effect models to test the impact of such financial aid on rehabilitation access.

**Results:**

The targeted financial aid significantly reduced the possibility of disability deterioration for elderly people with severe disabilities (0.26%; *P* < 0.001). Using PSM-DID models, the impact remained significant (0.33%; *P* < 0.001). Moreover, financial aid was significantly related to their access to rehabilitation services (12.71%; *P* < 0.001). Further analysis showed the heterogenous effects of targeted financial aid across individual demographic and socioeconomic factors, as well as communities with and without rehabilitation facilities.

**Conclusions:**

Targeted financial aid had a positive impact on preventing disability deterioration among elderly individuals aged ≥65 years with severe disabilities. Moreover, rehabilitation care had a potential mediating role in the relationship between targeted financial aid and disability deterioration prevention. This study highlights the effectiveness of targeted financial aid in preventing disability deterioration and improving rehabilitation care for people with disabilities.

With the significant extension of life expectancy, disability has been a major health threat to the ageing population in both developed countries as well as low- and middle-income countries (LMICs) [1,2]. More than 46% of people aged ≥60 years have disabilities, and more than 250 million elderly people experience disabilities around the world [3]. Disability in the ageing population impacts their quality of life, their families, as well as the entire health care system [4]. Elderly people with disabilities often have shortened lifespans, higher risks of developing comorbid conditions, and face more challenges in accessing health care services compared to their non-disabled counterparts [5–7]. This vulnerability is particularly salient in LMICs, which hold more than 80% of the population with disabilities worldwide [8]. Moreover, the deterioration of disabilities contributes to additional health problems [9] and is associated with elderly people’s quality of daily life and mental health status [10,11]. Therefore, addressing the problem of disabilities and disability deterioration is a key task for the realisation of healthy ageing around the world [12].

Targeted financial aid has been employed as a significant tool to assist people with disabilities and plays a pivotal role in enhancing their social protection [13]. For example, South Korea’s disability-targeted cash transfer programme was associated with increased inpatient care utilisation and reduced financial burdens related to health care expenditures [14]. Similarly, disability-targeted cash transfers in China improved access to rehabilitation services for children and adolescents living with disabilities [15]. In the USA, federal disability benefits were related to increased health care utilisation [16], and disability pensions in Sweden were associated with improved accessibility to psychiatric health care [17]. Conditional cash transfers in Brazil improved leprosy multidrug therapy adherence and cure rates in multibacillary cases [18]. Notably, the positive effects of disability-targeted financial aid can persist after the programme terminates without creating dependency or reducing labour supply [19,20].

Prior studies overlooked the effects of financial aid on disability deterioration for an ageing population. It was found that cash transfers were positively associated with improved disability status and increased access to disability-related services for adults with disabilities in China [21]. In the USA, supplemental security income was found to improve the mental well-being of people with substance abuse disabilities [20]. Denmark’s disability pension was related to lower odds of musculoskeletal disorders among beneficiaries [22]. Disability benefits in Norway were associated with reduced mental symptoms [23], and in the Netherlands, disability-targeted cash transfers were even associated with reduced mortality among people with disabilities [24]. However, few studies have focused on the impact of targeted financial aid on disability deterioration, which is a major health threat for the ageing population around the world [25]. Besides, the mechanism behind the impact of targeted financial has seldom been investigated. Such research gaps hinder the realisation of successful ageing, especially in LMICs.

Therefore, evaluating large-scale financial aid using population-level data and a quasi-experiment design is important to filling the above literature gap and providing policy implications. In 2016, the State Council of China, as the Chinese central government, launched a targeted financial aid programme aimed at people with life difficulties or severe disabilities, addressing immediate needs such as additional living expenses and long-term care expenses [26]. By July 2023, this programme had covered over 11 million people with disabilities in the Chinese mainland [27]. In this article, we aimed to examine the effectiveness of such financial aid on disability deterioration among the ageing population with disabilities in China, based on a quasi-experiment research design and a national administrative data set. Moreover, we aimed to test the heterogeneity and potential mechanism for the causal impact of financial aid on the ageing population with disabilities.

## METHODS

### Data

We collected the initiation time of targeted financial aid for people with severe disabilities in prefectural cities in China. Between 2016–19, three cities (Zhangjiakou, Qujing, and Chuzhou) never adopted a financial aid policy. At the same time, four cities (Leshan, Nanchang, Huainan, and Yulin) implemented targeted financial aid in 2018. In this study, the treatment group consisted of individuals in four cities who adopted the targeted financial aid in 2018, while the control group consisted of their counterparts in three cities without such aid between 2016–19. Thus, counterfactual differences can be estimated between the control group (in Zhangjiakou, Qujing, and Chuzhou) and the treatment group (in Leshan, Nanchang, Huainan, and Yulin).

Individual-level data utilised in this study were extracted from a cohort survey on people with disabilities in China. The survey collected information on respondents’ demographic characteristics, socioeconomic status, disability status, and access to rehabilitation services. Additionally, it documented individuals’ disability types (including visual, hearing, speech, physical, intellectual, psychiatric, and multiple disabilities). The survey also covered information on their residential communities, including disability rehabilitation facilities within communities.

**Figure 1:**
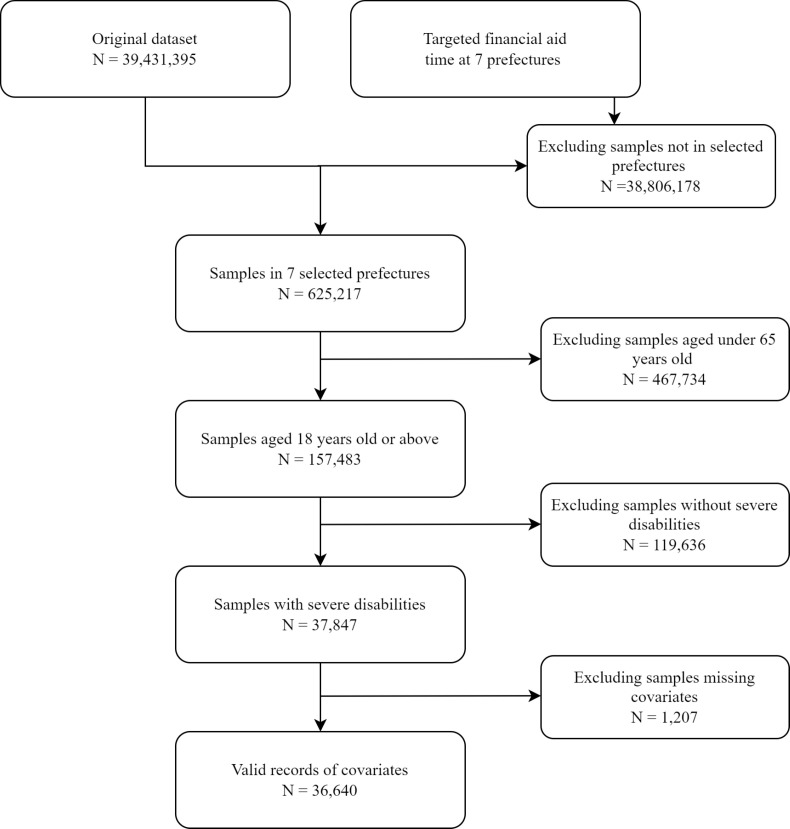
Flowchart of the study sample.

To identify the impact of targeted financial aid on the ageing population with disabilities, we identified each individual with the timing of targeted financial aid at the prefectural level. We also included community information covered in the same survey in our data set. First, we excluded 38 806 178 individuals from cities not covered in this study. Given the study’s focus on the elderly population, we excluded 467 734 individuals aged <65 years, the threshold used by the United Nations (UN) [28]. Further exclusions comprised 119 936 individuals whose disabilities were not severe, which rendered them ineligible for the targeted financial aid. Finally, 1207 individuals lacked records of covariates, including education level, residence, poverty status, marital status, and health insurance. We excluded them to reduce bias caused by missingness that is not random. The final data set included 36 640 individuals and 146 560 person-year observations (**Figure 1**).

### Disability deterioration and rehabilitation care

As the deterioration of disability contributes to additional health problems, such as quality of daily life and mental health status, to elderly people [9–11], disability deterioration is a key health threat to the ageing population. Therefore, we focused on disability multiplicity as the primary outcome to assess the deterioration of disabilities among elderly people with disabilities. In this study, we defined disability multiplicity as having multiple types rather than a single type of disability [29]. We coded respondents who reported having multiple disabilities as ‘1’ while single disability respondents as ‘0’. The transition from single to multiple disabilities denoted the deterioration of disability conditions for ageing people.

Moreover, we assumed that rehabilitation care is a potential mechanism to mitigate disability deterioration under targeted financial aid. The secondary outcome focused on rehabilitation care for elderly people with disabilities. It was measured by the access to rehabilitation services. The rehabilitation services encompassed surgeries, drugs, functional training, assistance devices, and caring aimed at respondents’ disabilities in the given year. We coded respondents who reported accessing rehabilitation services as ‘1’ and those who did not access rehabilitation services as ‘0’.

### Covariates

Besides public policies, the effects of targeted financial aid can be influenced by recipients’ demographic characteristics, residence locations, and the nature of their disability. For instance, previous research has shown that disability-targeted cash transfers were less utilised by women, older individuals, and individuals residing in remote areas [30]. Thus, in this study, we included a set of covariates to account for their potential confounding effects on respondents’ disability deterioration and rehabilitation care [15,31]. Demographic characteristics encompassed age, which was categorised into three age groups, using thresholds following a prior study in China [32], as well as gender. Socioeconomic status variables, categorised as in the survey questionnaire, included education (categorised as never educated, primary, middle school, high school, college or higher) and residence (classified as rural or urban). Additionally, health insurance coverage and marital status (classified as single, married, divorced, or widowed) were considered covariates due to their potential influence on elderly people with disabilities.

### Difference-in-differences analysis

In our primary analysis, we used difference-in-differences (DID) to assess the impact of targeted financial aid (implemented in 2018) on disability deterioration. This involved comparing the outcomes of respondents in prefectures where the policy was implemented with those in prefectures where it was not implemented. The treatment individuals were those with the implementation of the targeted financial aid policy at the prefectural level. Four prefectures applied the targeted financial aid for people with disabilities in 2018, while three did not adopt any such policy between 2016–19. Thus, counterfactual differences between the treatment and control groups can be estimated. The average treatment effect on the treated for elderly people with severe disabilities living in the four prefectures that adopted a targeted financial aid policy can be found. Importantly, this policy was not influenced by individual-level characteristics of people with disabilities, making it plausibly exogenous to individual disability conditions and rehabilitation access [33]. This DID design allowed a causal analysis of the policy’s impact [34,35]. As the difference in adoption dates of targeted financial aid at the prefectural level was unintended instead of selective, our ethical review recognised that this issue did not involve ethical problems. Besides, all prefectures covered in this study had adopted the aid policy by 2023.

We estimated linear probability models and controlled for covariates at the individual level. Fixed effects for each individual were included to account for measured and unmeasured time-invariant differences, and fixed effects for each year were incorporated to control for common trends in outcomes for all individuals. Robust standard errors clustered at the individual level were used. Covariates comprised individuals’ age, gender, education, residence, health insurance, and marital status. The main DID models can be expressed as follows:

*Y_it_* = *β_1_ *+ *β_1_A_it_* + *μX_it_* + *λ_i_* + *π_t_* + *ϵ_it_*

*Y_it_* denotes the outcome variables in this study (disability multiplicity) for individual *i* in year *t*. *β_0_* is the intercept, and *A_it_* refers to the targeted financial aid for individual *i* in year *t*. *β_1_* estimates the targeted financial aid for the average treatment effect on the treated. *X_it_* denotes all other covariates. *μ* is a set of parameters to be estimated. *λ_i_* are individual fixed effects, *π_t_* are year fixed effects, and *ϵ_it_* is the error term.

We assessed the validity of the DID method by relying on the parallel trend assumption, which suggests that trends in outcomes for respondents in policy-implemented prefectures and non-policy-implemented prefectures should be similar in the absence of the targeted financial aid policy. To verify this, we visually presented the outcomes trend for individuals in the treatment and control groups.

To further mitigate selective bias in the DID analysis and enhance the robustness of our results, we performed a propensity score matching-DID (PSM-DID) analysis. This method balances the distribution of covariates between treated and control groups by matching treated observations with control observations based on their propensity scores for residing in policy-implemented prefectures [36]. We first estimated the propensity score, considering individuals’ age, education, gender, residence, health insurance, marriage, and poverty status. Subsequently, each treated observation was matched with control observations from other units to align variations in covariates between treated and control groups. A calliper of 0.05 was used with a 1:2 ratio. The balance test result was significant, with a *P*-value <0.001, confirming the effectiveness of the matching process. Finally, we conducted the DID analysis using the matched samples to evaluate the causal impact of the targeted financial aid policy on disability deterioration, accounting for the matched covariate distributions.

For the secondary outcome, we used a fixed effects model to test the association between targeted financial aid and rehabilitation care for ageing people with disabilities. We estimated linear probability models and controlled for covariates at the individual level, fixed effects for each individual, and fixed effects for each year. Covariates comprised individuals’ age, gender, education, residence, health insurance, marital status, disability type and disability level. Prior studies showed that disadvantaged individuals face additional health disadvantages in terms of socioeconomic status and social service access [15,31].

To test the potential of targeted financial aid in addressing inequalities within people with disabilities, we also conducted a heterogeneity analysis for our two outcome variables across individuals’ and community features.

We used Stata, version 17.0 (StataCorp LLC, College Station, Texas, USA) for all descriptive analysis and statistical models. Statistical tests were two-sided, and the significant level of *P*-value <0.05.

### Robustness tests

We conducted supplementary analyses on alternative samples to ensure the robustness of our main findings. We analysed each of the four policy-implemented prefectures (Leshan, Nanchang, Huainan, and Yulin) within the treatment group. This allowed us to assess the consistency of the policy’s impact across different regions, providing additional insights into its effectiveness.

## RESULTS

### Sample description

The final data set comprised 36 640 ageing individuals and 146 560 person-year observations between 2016–19 in mainland China. Descriptive statistics of the sample are detailed in **Table 1**. For each year, the control group contained 8137 individuals, while the treatment group comprised 28 503 individuals. The trend plots of the primary outcome, disability multiplicity, were paralleled between the treatment and control groups leading up to the treatment in 2018 (Figure S1 in the **Online Supplementary Document**). While it is not feasible for us to test for similar trends well before the policy shock, our DID analysis remains valid as this approach is consistent with prior studies that used similar exogenous policy shocks in DID analyses [35,37].

**Table 1:** Sample description*

	Year							
**Items**	**2016**		**2017**		**2018**		**2019**	
	**Control†**	**Treatment‡**	**Control†**	**Treatment‡**	**Control†**	**Treatment‡**	**Control†**	**Treatment‡**
Age in years, x̄ (SD)	73.71 (6.48)	73.67 (6.73)	74.71 (6.48)	74.67 (6.73)	75.71 (6.48)	75.67 (6.73)	76.71 (6.48)	76.67 (6.73)
Gender								
*Female*	4638 (57.00)	15 016 (52.68)	4638 (57.00)	15 016 (52.68)	4638 (57.00)	15 016 (52.68)	4638 (57.00)	15 016 (52.68)
*Male*	3499 (43.00)	13 487 (47.32)	3499 (43.00)	13 487 (47.32)	3499 (43.00)	13 487 (47.32)	3499 (43.00)	13 487 (47.32)
Education								
*Illiterate*	5519 (67.83)	11 398 (39.99)	5483 (67.38)	11 270 (39.54)	4951 (60.85)	10 877 (38.16)	4590 (56.41)	10 557 (37.04)
*Primary school*	1842 (22.64)	13 730 (48.17)	1864 (22.91)	13 801 (48.42)	2114 (25.98)	13 976 (49.03)	2265 (27.84)	14 091 (49.44)
*Middle school*	646 (7.94)	2706 (9.49)	647 (7.95)	2766 (9.70)	893 (10.97)	2939 (10.31)	1066 (13.10)	3100 (10.88)
*Senior school*	105 (1.29)	532 (1.87)	116 (1.43)	527 (1.85)	152 (1.87)	563 (1.98)	183 (2.25)	578 (2.03)
*College or above*	25 (0.31)	137 (0.48)	27 (0.33)	139 (0.49)	27 (0.33)	148 (0.52)	33 (0.41)	177 (0.62)
Residence								
*Urban*	891 (10.95)	6200 (21.75)	883 (10.85)	6275 (22.02)	849 (10.43)	6240 (21.89)	855 (10.51)	6301 (22.11)
*Rural*	7246 (89.05)	22 303 (78.25)	7254 (89.15)	22 228 (77.98)	7288 (89.57)	22 263 (78.11)	7282 (89.49)	22 202 (77.89)
Poverty status								
*Not poverty*	3453 (42.44)	16 864 (59.17)	4207 (51.70)	17 547 (61.56)	4169 (51.24)	17 010 (59.68)	5277 (64.85)	19 195 (67.34)
*Poverty*	4684 (57.56)	11 639 (40.83)	3930 (48.30)	10 956 (38.44)	3968 (48.76)	11 493 (40.32)	2860 (35.15)	9308 (32.66)
Marriage								
*Single*	562 (6.91)	1650 (5.79)	526 (6.46)	1553 (5.45)	746 (9.16)	1715 (6.02)	536 (6.59)	1498 (5.26)
*Married*	5444 (66.90)	20 239 (71.01)	5647 (69.4)	20 284 (71.16)	4870 (59.85)	19 372 (67.96)	5600 (68.82)	20 113 (70.56)
*Divorced*	62 (0.76)	237 (0.83)	284 (3.49)	360 (1.26)	91 (1.11)	262 (0.92)	422 (5.19)	539 (1.89)
*Widowed*	2069 (25.43)	6377 (22.37)	1680 (20.65)	6306 (22.12)	2430 (29.86)	7154 (25.10)	1579 (19.41)	6353 (22.29)
Health insurance								
*Not have*	419 (5.15)	3169 (11.12)	484 (5.95)	2883 (10.11)	640 (7.87)	2830 (9.93)	483 (5.94)	2420 (8.49)
*Have*	7718 (94.85)	25 334 (88.88)	7653 (94.05)	25 620 (89.89)	7497 (92.13)	25 673 (90.07)	7654 (94.06)	26 083 (91.51)
Disability level								
*Level 1*	6110 (75.09)	18 635 (65.38)	6104 (75.02)	18 599 (65.25)	6103 (75.00)	18 561 (65.12)	6086 (74.79)	18 522 (64.98)
*Level 2*	2027 (24.91)	9868 (34.62)	2033 (24.98)	9904 (34.75)	2034 (25.00)	9942 (34.88)	2051 (25.12)	9981 (35.02)
Disability type								
*Visual*	2058 (25.29)	8364 (29.34)	2057 (25.28)	8361 (29.33)	2056 (25.27)	8359 (29.33)	2051 (25.21)	8369 (29.36)
*Hearing*	672 (8.26)	3401 (11.93)	664 (8.16)	3403 (11.94)	662 (8.14)	3402 (11.94)	654 (8.04)	3451 (12.11)
*Speech*	113 (1.39)	576 (2.02)	113 (1.39)	576 (2.02)	113 (1.39)	575 (2.02)	106 (1.30)	567 (1.99)
*Physical*	4183 (51.41)	12 708 (44.58)	4182 (51.39)	12 700 (44.56)	4180 (51.37)	12 696 (44.54)	4176 (51.32)	12 665 (44.43)
*Intellectual*	170 (2.09)	460 (1.61)	169 (2.08)	460 (1.61)	168 (2.06)	459 (1.61)	166 (2.04)	449 (1.58)
*Psychiatric*	349 (4.29)	2084 (7.31)	349 (4.29)	2085 (7.32)	349 (4.29)	2081 (7.30)	348 (4.28)	2074 (7.28)
*Multiple disabilities*	592 (7.28)	910 (3.19)	603 (7.41)	918 (3.22)	609 (7.48)	931 (3.27)	636 (7.82)	928 (3.26)
Rehabilitation access								
*No*	5229 (64.26)	22 919 (80.41)	5446 (66.93)	18 024 (64.64)	5587 (68.66)	19 103 (67.02)	6497 (79.85)	19 693 (69.09)
*Yes*	2908 (35.74)	5584 (19.59)	2691 (33.07)	10 079 (35.36)	2550 (31.34)	9400 (32.98)	1640 (20.15)	8810 (30.91)

SD – standard deviation, x̄ – mean

*Presented as n (%) unless specified otherwise.

†N = 8137.

‡N = 28 503.

### Primary analysis

First, we focused on the impact of targeted financial aid on disability deterioration for elderly people. The findings indicated that following implementation in 2018 for the treatment group, targeted financial aid decreased the 0.26% likelihood (*P* < 0.001) of having multiple disabilities compared to having a single-type disability, controlling for other covariates, disability levels, and individual and time-fixed effects. In essence, targeted financial aid had a significant preventive effect on disability deterioration for individuals (**Table 2**).

**Table 2:** Primary outcome results

	Disability multiplicity					
	**DID**			**PSM-DID**		
Outcomes*	**Model 1**	**Model 2**	**Model 3**	**Model 4**	**Model 5**	**Model 6**
DID (%)	–1.5	–0.25	–0.26	–2.72	–0.33	–0.33
*Robust SE*	0.11	0.06	0.06	0.31	0.08	0.08
*P value*	<0.001	<0.001	<0.001	<0.001	<0.001	<0.001
Adjusted R-squared	0.001	0.957	0.968	0.001	0.972	0.973
Covariates†	No	No	Yes	No	No	Yes
Individual fixed effects	No	Yes	Yes	No	Yes	Yes
Year fixed effects	No	Yes	Yes	No	Yes	Yes
Cities (n)	7	7	7	7	7	7
Individuals (n)	36 640	36 640	36 640	36 364	35 913	35 913
Observations (n)	146 560	146 560	146 560	140 630	140 179	140 179

DID – difference-in-differences, PSM-DID – propensity score matching-DID, SE – standard error.

*Outcomes in all models were multiplied as 100% scales.

†Including age (log), gender, education, residence, health insurance, marital status, and disability level.

**Figure 2:**
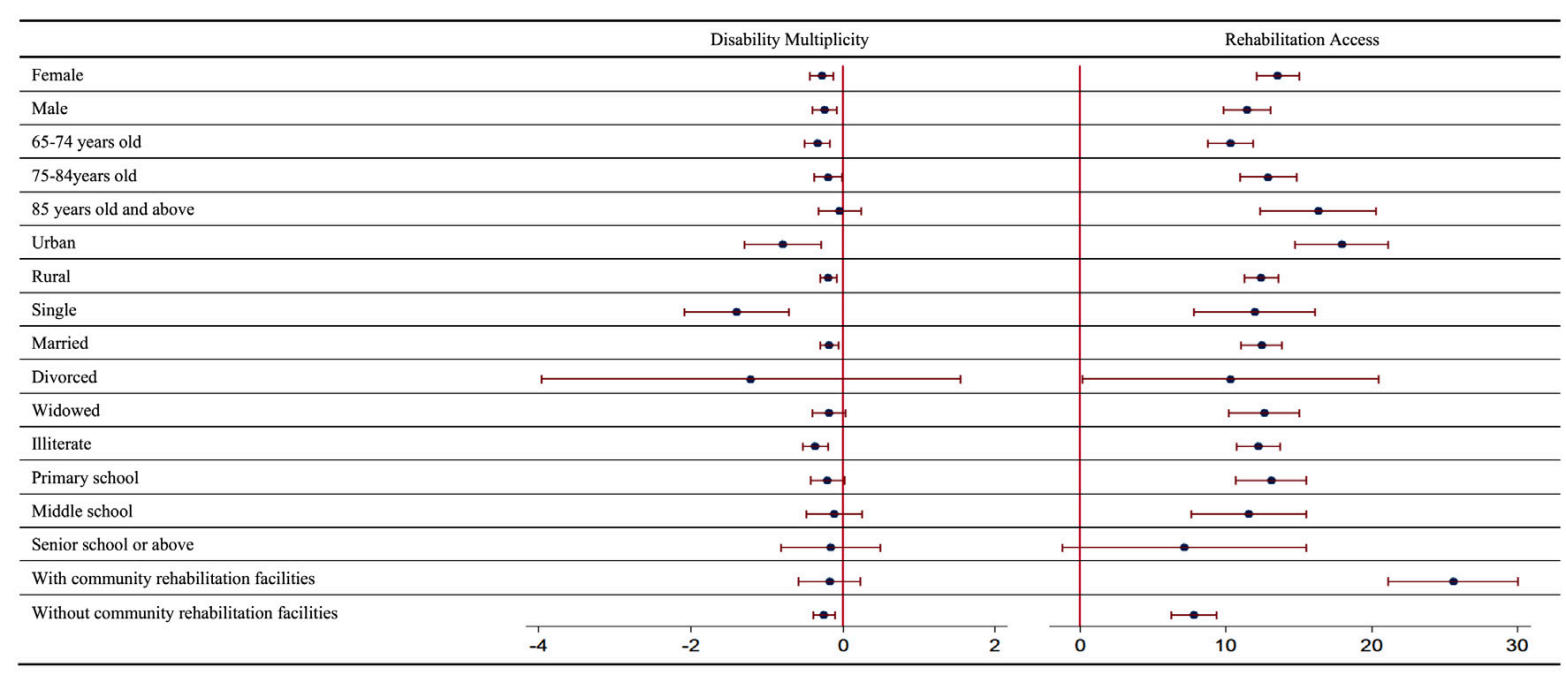
Heterogeneity analysis results.

Like other assistance measures, the impact of targeted financial aid is influenced by individual and community characteristics. Targeted financial aid had a stronger effect on reducing disability multiplicity for female individuals (–0.28%; *P* < 0.001) compared to male individuals (–0.24%; *P* < 0.001) in the treatment group. The effect of targeted financial aid on reducing disability multiplicity was strongest among the youngest age group (–0.33%; *P* < 0.001). Further, targeted financial aid had a stronger impact on reducing disability multiplicity among urban residents (–0.78%; *P* = 0.002) than among rural residents (–0.19%; *P* = 0.001). The effect of targeted financial aid on reducing disability multiplicity was strongest for single individuals (–1.39%; *P* < 0.001). The effect of targeted financial aid on reducing disability multiplicity was significant among the least educated group (–0.36%; *P* < 0.001) but not significant for individuals with higher education levels (for primary school *P* = 0.080; for middle school *P* = 0.540; for senior school and above *P* = 0.673) (**Figure 2**). Additionally, we explored the heterogeneous effects of targeted financial aid in communities with and without rehabilitation facilities for people with disabilities. The effect of targeted financial aid on reducing disability multiplicity was only significant when communities did not have rehabilitation facilities (–0.24%; *P* = 0.001).

### Propensity score matching

We employed PSM-DID models to mitigate selective bias and enhance the robustness of our estimations. Targeted financial aid exhibited consistent effects, reducing the likelihood of having multiple disabilities rather than a single disability by 0.33% (*P* < 0.001) (**Table 2**). The results of sensitivity analysis for PSM-DID using alternative ratios and calibres yielded consistent results (Table S1 in the **Online Supplementary Document**). These findings underscore the robustness and effectiveness of targeted financial aid in mitigating disability deterioration for elderly people with severe disabilities.

### Secondary analysis

In addition to the primary analysis, we explored the potential mechanism behind the impact of targeted financial aid as we investigated the association between targeted financial aid and rehabilitation care for elderly individuals with severe disabilities. The results revealed that targeted financial aid was associated with a higher likelihood to access rehabilitation services by 12.71% on average (*P* < 0.001) for individuals in the treatment group after adjusting for covariables, disability level, disability type, and individual and time-fixed effects (**Table 3**).

**Table 3:** Secondary outcome results

	Rehabilitation access		
Outcomes*	**Model 1**	**Model 2**	**Model 3**
Targeted financial aid (%)	3.52	13.13	12.71
*Robust SE*	0.23	0.55	0.56
*P value*	<0.001	<0.001	<0.001
Adjusted R-squared	0.001	0.329	0.33
Covariates†	No	No	Yes
Individual fixed effects	No	Yes	Yes
Year fixed effects	No	Yes	Yes
Cities (n)	7	7	7
Individuals (n)	36 640	36 640	36 640
Observations (n)	146 560	146 560	146 560

SE – standard error

*Outcomes in all models were multiplied as 100% scales

†Including age (log), gender, education, residence, health insurance, marital status, and disability level.

The role of rehabilitation care also varied with individual and community features. **Figure 2** shows the heterogeneous associations between targeted financial aid and rehabilitation care. First, the associations were stronger among females (13.59%; *P* < 0.001) than males (11.48%; *P* < 0.001). Second, the associations increased from the youngest (10.34%; *P* < 0.001) to the oldest age group (16.36%; *P* < 0.001). Third, the associations were stronger for urban residents (17.96%; *P* < 0.001) than for rural residents (12.43%; *P* < 0.001). Fourthly, the associations were strongest among widowed individuals (12.65%; *P* < 0.001). Fifthly, the associations were strongest for the least educated group (12.22%; *P* < 0.001). Lastly, the associations were significantly stronger in communities with rehabilitation facilities (25.63%; *P* < 0.001) than in communities without such facilities (7.82%; *P* < 0.001).

### Robustness tests

To ensure the robustness of the main findings, we conducted supplementary analyses on alternative samples, focusing specifically on each of the four prefectures where the targeted financial aid policy was implemented (Leshan, Nanchang, Huainan, and Yulin). The results were consistent with the main sample (Tables S2–3 in the **Online Supplementary Document**).

## DISCUSSION

Based on an administrative cohort data set and employing the DID causal inference method, in this study, we found that targeted financial aid had a positive effect on disability deterioration prevention for elderly people with severe disabilities, and results from PSM-DID analysis corroborated DID results. We found that targeted financial aid significantly reduced the possibility of elderly people with disabilities in the treatment group developing multiple disabilities. Moreover, the heterogeneity analysis revealed that targeted financial aid was particularly likely advantageous for vulnerable ageing subpopulations, underscoring their potential to reduce health inequalities among elderly people with disabilities. Additionally, targeted financial aid was associated with enhanced access to rehabilitation services for elderly people with severe disabilities. By preventing multiple disabilities, such targeted financial aid contributes to reducing the likelihood of secondary conditions that could exacerbate overall health conditions among individuals with disabilities [21,38–40].

The psychological burden on individuals with disabilities tends to escalate with age, often accompanied by an increased incidence of conditions like stroke and dementia [41,42]. This study’s findings showed that compared to their younger counterparts, people with disabilities aged ≥85 years benefited more from targeted financial aid regarding rehabilitation access. However, they exhibited comparatively less in preventing disability deterioration. Therefore, we suggest that tailored policies are needed that specifically address the unique needs and challenges faced by people aged 85 or above with disabilities. These policies should consider the distinct characteristics and vulnerabilities of this group of people with disabilities to ensure comprehensive support and improved outcomes under targeted financial aid.

Notably, our results show that the targeted financial aid had a stronger impact on the ageing population with disabilities living in urban areas compared to their counterparts living in rural areas. This could be explained by the relatively poorer provision of health care facilities and professionals in rural areas, which are potential proxies of financial aid measures, compared to urban areas in China [31]. Our results also demonstrate that the effects of targeted financial aid on the ageing population with effects vary across community features. The association between targeted financial aid and access to rehabilitation services was stronger for elderly people living in communities with rehabilitation facilities than those without such facilities. However, the DID results show that the impact of targeted financial aid on disability deterioration was significant for elderly people living in communities without rehabilitation facilities, suggesting that individualised financial aid made up for the absence of community care. Therefore, it is important to introduce individualised social assistance programs for elderly people where community and regional social services are underdeveloped.

This study’s findings highlight the practical significance of targeted financial programmes [43,44], particularly in LMICs where the implementation of financial aid policies is often insufficient [45]. Despite increased social security measures, socioeconomically disadvantaged people with disabilities tend to have restricted access to health care and rehabilitation services [15,31]. Our results showed that targeted financial aid prevented disability deterioration and improved rehabilitation access for disadvantaged groups, such as females or those with lower education levels. Thus, beyond reducing overall inequality between people with and without disabilities, targeted financial aid programmes have the potential to mitigate disparities between different subgroups of people with disabilities.

We also underscore the importance of rehabilitation services for people with disabilities in the context of financial aid policies. This study revealed that targeted financial aid was significantly associated with improved access to rehabilitation services for the ageing population with disabilities. Moreover, prior evidence indicated that rehabilitation helps reduce the severity of disabilities [9,46,47]. Although in this study we did not prove the mechanism directly, these findings jointly suggested the mediation role of rehabilitation services in improving the health status of an ageing population with disabilities. Thus, improving rehabilitation access for people with disabilities is a key measure to improving the health status of this population [48,49]. Our analysis also demonstrated the heterogeneous effects of targeted financial aid across residential communities with and without rehabilitation facilities. Thus, in addition to financial aid programmes, community-level public service facilities should also be included in disability policies.

The study has several limitations that should be acknowledged. First, we only focus on the ageing population in China. The effects of targeted financial aid on ageing populations with disabilities in developed countries and other LMICs require further investigation to address ageing with disabilities worldwide. Thus, caution should be taken when generalising findings in this study to other LMICs with different health care and social welfare systems and demographic structures. Second, due to the study’s short duration, it was difficult to establish parallel trend assumptions and account for temporal changes over longer periods, which could affect the robustness of the findings [50]. Third, the absence of records for certain potential confounding variables such as health behaviours, chronic diseases, and income level limits the validity of the analysis. For example, as prior evidence shows that smoking, diabetes, and low income lead to a higher risk of developing disability [51–53], ignoring such risk effects could lead to a biased estimation of the impact of targeted financial aid. Fourth, although we conducted a sensitivity analysis for PSM-DID using alternative ratios and calibres, unobserved confounders could still lead to unbalances between control and treatment groups. Thus, to mitigate these limitations, future studies could use more comprehensive data covering other countries or longer periods, or conduct randomised experiments to eliminate biases caused by potential confounders.

Albeit these limits, this study contributes to understanding targeted financial aid in several ways. First, it provides a quasi-experimental framework for testing the causal effects of targeted financial aid on disability deterioration, a major threat to the health of the ageing population worldwide. Second, by conducting a heterogeneity analysis, it explores variations in targeted financial aid’s effects on individuals across various demographic and socioeconomic factors and community characteristics. Third, the study explores the impact of targeted financial aid on access to rehabilitation services, suggesting a potential intermediary role of rehabilitation in mitigating disability deterioration, which emphasises the potential of social services to act as mediators between targeted financial aid and the ageing population.

## CONCLUSIONS

Our results showed that a targeted financial aid policy in China has reduced the possibility of elderly people with disabilities developing multiple disabilities. Moreover, our heterogeneity analysis indicated that targeted financial aid was particularly likely advantageous for vulnerable subpopulations without people with disabilities, which underscored the policy’s potential to reduce health inequalities among elderly people with disabilities. In addition, we found that targeted financial aid was associated with an improved likelihood of accessing rehabilitation services for benefited elderly people with severe disabilities. Our results highlight the effectiveness of targeted financial policies in practice, particularly in LMICs where general social assistance is often insufficient. We also underscore the significant role of rehabilitation services in improving health outcomes for people with disabilities.

## Additional material


Online Supplementary Document

## References

[1] Tesch-Römer C Wahl HW Toward a More Comprehensive Concept of Successful Aging: Disability and Care Needs. J Gerontol B Psychol Sci Soc Sci. 2017;72:310. 27988482 10.1093/geronb/gbw162 PMC5926993

[2] Zheng X Chen G Song X Liu J Yan L Du W Twenty-year trends in the prevalence of disability in China. Bull World Health Organ. 2011;89:788–97. 10.2471/BLT.11.089730 22084524 PMC3209727

[3] United Nations. Ageing And Disability. 2024. Available: https://social.desa.un.org/issues/disability/disability-issues/ageing-and-disability#:~:text=More%20than%2046%20per%20cent%20of%20older,likely%20to%20lead%20to%20further%20increases%20in. Accessed: 1 May 2024.

[4] Guralnik JM Fried LP Salive’ ME Disability as a public health outcome in the aging population. Annu Rev Public Health. 1996;17:25–46. 10.1146/annurev.pu.17.050196.000325 8724214

[5] Iezzoni LI Eliminating Health And Health Care Disparities Among The Growing Population Of People With Disabilities. Health Aff (Millwood). 2011;30:1947–54. 10.1377/hlthaff.2011.0613 21976339

[6] World Health Organization. Sitora’s story: building a better future for people with disability. 2019. Available: https://www.who.int/news-room/feature-stories/detail/sitora-s-story-building-a-better-future-for-people-with-disability. Accessed: 1 May 2024.

[7] World Health OrganizationDisability. 2024. Available: https://www.who.int/health-topics/disability#tab=tab_1. Accessed: 1 May 2024.

[8] Thota AB, Mogo ERI, Igbelina DC, Sheaf GS, Mustafa R, Bakrania S, et al. Effectiveness of Inclusive Interventions for Children with Disabilities in Low-and Middle-Income Countries: Protocol for an Evidence and Gap Map. Florence, Italy: UNICEF Office of Research; 2022. Available: https://www.unicef.org/innocenti/media/4721/file/UNICEF-Effectiveness-Inclusive-Interventions-Children-Disabilities-2022.pdf. Accessed: 16 October 2024.

[9] Rimmer JH Health Promotion for People With Disabilities: The Emerging Paradigm Shift From Disability Prevention to Prevention of Secondary Conditions. Phys Ther. 1999;79:495–502. 10.1093/ptj/79.5.495 10331753

[10] Baumstarck K Pelletier J Butzkueven H Fernández O Flachenecker P Idiman E Health-related quality of life as an independent predictor of long-term disability for patients with relapsing–remitting multiple sclerosis. Eur J Neurol. 2013;20:907. 10.1111/ene.12087 23347258

[11] Reynolds SL Silverstein M Observing the onset of disability in older adults. Soc Sci Med. 2003;57:1875–89. 10.1016/S0277-9536(03)00053-4 14499512

[12] Rowe JW Kahn RL Successful Aging 2.0: Conceptual Expansions for the 21st Century. J Gerontol B Psychol Sci Soc Sci. 2015;70:593–6. 10.1093/geronb/gbv025 25878054

[13] Ziebold C Paula CS Santos IS Barros FC Munhoz TN Lund C Conditional cash transfers and adolescent mental health in Brazil: Evidence from the 2004 Pelotas Birth Cohort. J Glob Health. 2021;11:04066. 10.7189/jogh.11.04066 34737866 PMC8564883

[14] Jeon B Noguchi H Kwon S Ito T Tamiya N Disability, poverty, and role of the basic livelihood security system on health services utilization among the elderly in South Korea. Soc Sci Med. 2017;178:175–83. 10.1016/j.socscimed.2017.02.013 28237862

[15] Wang H Li Z Chen S Qin W Xie L Kong Y The effect of a disability-targeted cash transfer program on universal health coverage and universal access to education: a nationwide cohort study of Chinese children and adolescents with disabilities. Lancet Reg Health West Pac. 2022;31:100635. 36879791 10.1016/j.lanwpc.2022.100635 PMC9985013

[16] Pellegrini LC Geissler KH Disability, Federal Disability Benefits, and Health Care Access After the Affordable Care Act. J Disabil Policy Stud. 2021;31:244–54. 10.1177/1044207320919959

[17] Rahman S Mittendorfer-Rutz E Alexanderson K Jokinen J Tinghög P Disability pension due to common mental disorders and healthcare use before and after policy changes; a nationwide study. Eur J Public Health. 2017;27:90. 10.1093/eurpub/ckx187.174 28177513

[18] Pescarini JM Williamson E Ichihara MY Fiaccone RL Forastiere L Ramond A Conditional Cash Transfer Program and Leprosy Incidence: Analysis of 12.9 Million Families From the 100 Million Brazilian Cohort. Am J Epidemiol. 2020;189:1547–58. 10.1093/aje/kwaa127 32639534 PMC7705605

[19] Mitra S Disability Cash Transfers in the Context of Poverty and Unemployment: The Case of South Africa. World Dev. 2010;38:1692–709. 10.1016/j.worlddev.2010.06.014

[20] Watkins KE Podus D Lombardi E Burnam A Changes in Mental Health and Service Use After Termination of SSI Benefits. Psychiatr Serv. 2001;52:1210–5. 10.1176/appi.ps.52.9.1210 11533395

[21] Li Z Wang H Chen S Kong Y Xie L Zhang X The association of a disability-targeted cash transfer programme with disability status and health-care access: a quasi-experimental study using a nationwide cohort of 4·3 million Chinese adults living with severe disabilities. Lancet Public Health. 2023;8:e933–42. 10.1016/S2468-2667(23)00215-3 38000888

[22] Borup LJ Weye NØ Jensen V Fonager K Healthcare use before and after changing disability pension policy: a regional Danish cohort study. Eur J Public Health. 2019;29:1068–73. 10.1093/eurpub/ckz107 31168632

[23] Øverland S Glozier N Henderson M Mæland JG Hotopf M Mykletun A Health status before, during and after disability pension award: the Hordaland Health Study (HUSK). Occup Environ Med. 2008;65:769–73. 10.1136/oem.2007.037861 18940958

[24] García-Gómez P Gielen AC Mortality effects of containing moral hazard: Evidence from disability insurance reform. Health Econ. 2018;27:606–21. 10.1002/hec.3617 29237234

[25] Ydreborg B Ekberg K Nordlund A Health, quality of life, social network and use of health care: A comparison between those granted and those not granted disability pensions. Disabil Rehabil. 2006;28:25–32. 10.1080/09638280500165179 16393830

[26] The State Council. Opinions of the State Council on the Comprehensive Establishment of Subsidies for the Living of Persons with Disabilities in Difficulties and Nursing Subsidies for Severe Disabilities. 2015. Available: https://www.gov.cn/zhengce/content/2015-09/25/content_10181.htmPlease. Accessed: 1 May 2024.

[27] China Women’s News. Different lives, the same splendor. 2023. Available: https://epaper.cnwomen.com.cn/html/2023-09/18/nbs.D110000zgfnb_2.htm. Accessed: 1 May 2024.

[28] United Nations. Ageing. 2024. Available: https://www.un.org/en/global-issues/ageing. Accessed: 1 May 2024.

[29] Sloper P Turner S Risk and Resistance Factors in the Adaptation of Parents of Children with Severe Physical Disability. J Child Psychol Psychiatry. 1993;34:167–88. 10.1111/j.1469-7610.1993.tb00978.x 8444991

[30] Hameed S Banks LM Usman SK Kuper H Access to the Disability Allowance in the Maldives: National coverage and factors affecting uptake. Glob Soc Policy. 2023;23:127–47. 10.1177/14680181221084854

[31] Zhao X Wang H Disparities in unmet health service needs among people with disabilities in China. Int J Qual Health Care. 2021;33:mzab136. 10.1093/intqhc/mzab136 34613344

[32] Hou C Ma Y Yang X Tao L Zheng D Liu X Disability Transitions and Health Expectancies among Elderly People Aged 65 Years and Over in China: A Nationwide Longitudinal Study. Aging Dis. 2019;10:1246. 10.14336/AD.2019.0121 31788336 PMC6844588

[33] Herd P Schoeni RF House JS Upstream Solutions: Does the Supplemental Security Income Program Reduce Disability in the Elderly? Milbank Q. 2008;86:5–45. 10.1111/j.1468-0009.2007.00512.x 18307476 PMC2690339

[34] Ramesh BM Dehury B Isac S Gothalwal V Prakash R Namasivayam V The contribution of district prioritization on maternal and newborn health interventions coverage in rural India. J Glob Health. 2020;10:010418. 10.7189/jogh.10.010418 32373334 PMC7182352

[35] Newton-Lewis TA Bahety G Evaluating the effectiveness of Community Health Worker home visits on infant health: A quasi-experimental evaluation of Home Based Newborn Care Plus in India. J Glob Health. 2021;11:04060. 10.7189/jogh.11.04060 34737860 PMC8542379

[36] Lichand G Doria CA Leal-Neto O Fernandes JPC The impacts of remote learning in secondary education during the pandemic in Brazil. Nat Hum Behav. 2022;6:1079–86. 10.1038/s41562-022-01350-6 35618779 PMC9391221

[37] Tiendrebeogo CO Joseph V Bicaba F Bila A Bicaba A Druetz T Does abolishing user fees for family planning increase contraception use? An impact evaluation of the national policy in Burkina Faso. J Glob Health. 2022;12:04086. 10.7189/jogh.12.04086 36227754 PMC9559360

[38] Banks E Byles JE Gibson RE Rodgers B Latz IK Robinson IA Is psychological distress in people living with cancer related to the fact of diagnosis, current treatment or level of disability? Findings from a large Australian study. Med J Aust. 2010;193:S62–7. 10.5694/j.1326-5377.2010.tb03931.x 21542449

[39] Molton IR Terrill AL Smith AE Yorkston KM Alschuler KN Ehde DM Modeling Secondary Health Conditions in Adults Aging With Physical Disability. J Aging Health. 2014;26:335–59. 10.1177/0898264313516166 24388897

[40] Wilber N Mitra M Walker DK Allen D Disability as a Public Health Issue: Findings and Reflections from the Massachusetts Survey of Secondary Conditions. Milbank Q. 2002;80:393–421. 10.1111/1468-0009.00009 12101878 PMC2690115

[41] Hsieh N Waite L Disability, Psychological Well-Being, and Social Interaction in Later Life in China. Res Aging. 2019;41:362–89. 10.1177/0164027518824049 30636536

[42] Han Y Hu K Wu Y Fang Y Future life expectancy with disability among elderly Chinese individuals: a forecast based on trends in stroke and dementia. Public Health. 2021;198:62–8. 10.1016/j.puhe.2021.06.013 34364000

[43] McGuire J Kaiser C Bach-Mortensen AM A systematic review and meta-analysis of the impact of cash transfers on subjective well-being and mental health in low- and middle-income countries. Nat Hum Behav. 2022;6:359-70. 10.1038/s41562-021-01252-z 35058643

[44] Richterman A Thirumurthy H The effects of cash transfer programmes on HIV-related outcomes in 42 countries from 1996 to 2019. Nat Hum Behav. 2022;6:1362–71. 10.1038/s41562-022-01414-7 35851840

[45] Jerwanska V Kebbie I Magnusson L Coordination of health and rehabilitation services for person with disabilities in Sierra Leone – a stakeholders’ perspective. Disabil Rehabil. 2023;45:1796–804. 10.1080/09638288.2022.2074551 35603804

[46] Costa-Black KM Cheng ASK Li M Loisel P The Practical Application of Theory and Research for Preventing Work Disability: A New Paradigm for Occupational Rehabilitation Services in China? J Occup Rehabil. 2011;21:S15–27. 10.1007/s10926-011-9296-2 21365303

[47] Qiu N Zhang T Cheng J Examining the impact of spatial accessibility to rehabilitation facilities on the degree of disability: A heterogeneity perspective. SSM Popul Health. 2023;23:101489. 10.1016/j.ssmph.2023.101489 37588767 PMC10425410

[48] Cieza A Causey K Kamenov K Hanson SW Chatterji S Vos T Global estimates of the need for rehabilitation based on the Global Burden of Disease study 2019: a systematic analysis for the Global Burden of Disease Study 2019. Lancet. 2021;396:2006–17. 10.1016/S0140-6736(20)32340-0 33275908 PMC7811204

[49] Negrini S Kiekens C Heinemann AW Özçakar L Frontera WR Prioritising people with disabilities implies furthering rehabilitation. Lancet. 2020;395:111. 10.1016/S0140-6736(19)32623-6 31806258

[50] Vie GÅ Pape K Krokstad S Johnsen R Bjørngaard JH Temporal changes in health within 5 years before and after disability pension–the HUNT Study. Eur J Public Health. 2017;27:653–9. 10.1093/eurpub/ckx082 28637220

[51] Broese van Groenou MI Deeg DJH Penninx BWJH Income differentials in functional disability in old age: Relative risks of onset, recovery, decline, attrition and mortality. Aging Clin Exp Res. 2003;15:174–83. 10.1007/BF03324497 12889850

[52] Artaud F Dugravot A Sabia S Singh-Manoux A Tzourio C Elbaz A Unhealthy behaviours and disability in older adults: Three-City Dijon cohort study. BMJ. 2013;347:f4240. 10.1136/bmj.f4240 23881930

[53] Wong E Backholer K Gearon E Harding J Freak-Poli R Stevenson C Diabetes and risk of physical disability in adults: a systematic review and meta-analysis. Lancet Diabetes Endocrinol. 2013;1:106–14. 10.1016/S2213-8587(13)70046-9 24622316

