# Supplementary material for: Effectiveness of targeted financial aid on disability welfare for the ageing population in China: A quasi-experiment study
Source: J Glob Health. 2024 Oct 25;14:04222. doi: 10.7189/jogh.14.04222 (PMC11512167; doi:10.7189/jogh.14.04222)
Supplement: Online Supplementary Document [file jogh-14-04222-s001.pdf]

Online Supplementary Document

Wang et al. Effectiveness of Targeted Financial Aid on Disability Welfare for Aging Population in China: A Quasi-Experiment Study

J Glob Health

Figure S1. Parallel Trend Plot.

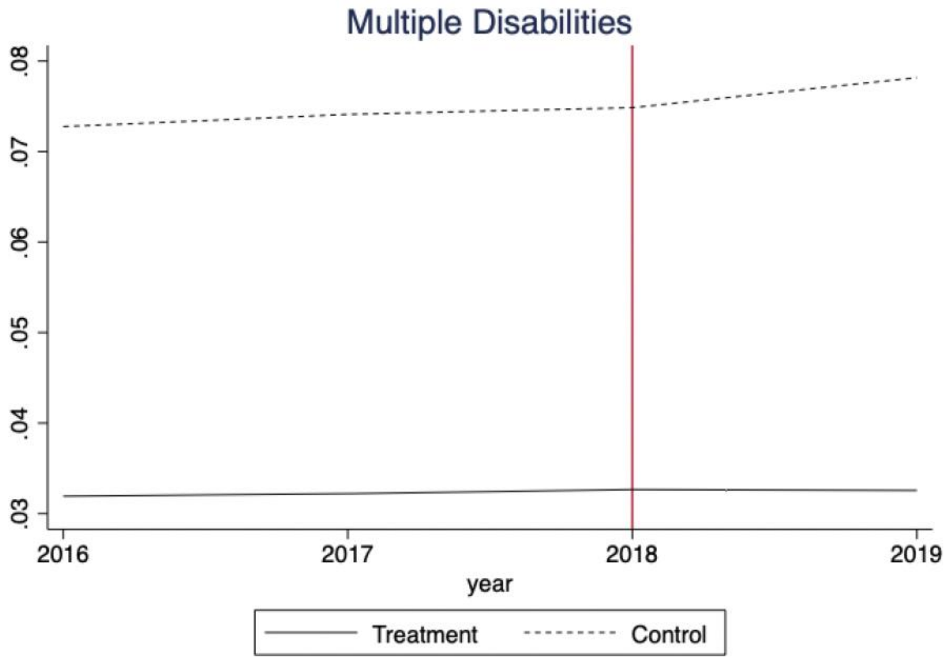

Table S1. Sensitivity Analysis for PSM-DID.

	Disability Multiplicity PSM-DID					
	(1)	(2)	(3)	(4)	(5)	(6)
DID	-0.333	-0.334	-0.333	-0.333	-0.346	-0.346
Robust SE	0.084	0.084	0.084	0.084	0.087	0.087
P value	<0.001	<0.001	<0.001	<0.001	<0.001	<0.001
Ratio	1:2	1:2	1:1	1:1	1:3	1:3
Caliber	0.05	0.03	0.05	0.03	0.05	0.03
Adjusted R-Squared	0.973	0.973	0.973	0.973	0.972	0.972
Number of cities	7	7	7	7	7	7
Number of individuals	35,913	35,913	35,886	35,886	35,967	35,967
Observations	140,179	140,179	139,980	139,980	140,435	140,435

Outcomes were multiplied as 100% scales. For precise comparison across models, results are kept in three-digit decimals.

Table S2. Alternative Sample Tests for Disability Multiplicity.

	Disability Multiplicity			
	(1)<br>Leshan	(2)<br>Nanchang	(3)<br>Huainan	(4)<br>Yulin
DID	-0.28	-0.15	-0.38	-0.36
Robust SE	0.06	0.06	0.09	0.22
P value	<0.001	0.019	<0.001	0.097
Adjusted R-Squared	0.954	0.981	0.974	0.980
Covariates*	Yes	Yes	Yes	Yes
Individual fixed effects	Yes	Yes	Yes	Yes
Year fixed effects	Yes	Yes	Yes	Yes
Number of cities	5	5	5	5
Number of individuals	27,505	13,400	11,646	8,500
Observations	110,020	53,600	46,584	34,000

Outcomes were multiplied as 100% scales.
* Including age (log), gender, education, residence, health insurance, marital status, and disability level.

Table S3. Alternative Sample Tests for Rehabilitation Access.

	Rehabilitation Access			
	(1) Leshan	(2) Nanchang	(3) Huainan	(4) Yulin
Targeted Financial Aid	17.04	4.63	3.14	11.54
Robust SE	0.58	0.73	0.83	2.03
P value	<0.001	<0.001	<0.001	<0.001
Adjusted R-Squared	0.335	0.231	0.239	0.193
Covariates*	Yes	Yes	Yes	Yes
Individual fixed effects	Yes	Yes	Yes	Yes
Year fixed effects	Yes	Yes	Yes	Yes
Number of cities	5	5	5	5
Number of individuals	27,505	13,400	11,646	8,500
Observations	110,020	53,600	46,584	34,000

Outcomes were multiplied as 100% scales.
* Including age (log), gender, education, residence, health insurance, marital status, and disability level.
